# One-Dimensional Nanostructures: Microfluidic-Based Synthesis, Alignment and Integration towards Functional Sensing Devices

**DOI:** 10.3390/s18010134

**Published:** 2018-01-05

**Authors:** Yanlong Xing, Petra S. Dittrich

**Affiliations:** 1 Leibniz-Institut für Analytische Wissenschaften—ISAS—e. V, 12489 Berlin, Germany; yanlong.xing@isas.de; 2 Department of Biosystems Science and Engineering, ETH Zürich, 4058 Basel, Switzerland

**Keywords:** one-dimensional nanostructures, microfluidics, continuous flow, alignment, nanosensor

## Abstract

Microfluidic-based synthesis of one-dimensional (1D) nanostructures offers tremendous advantages over bulk approaches e.g., the laminar flow, reduced sample consumption and control of self-assembly of nanostructures. In addition to the synthesis, the integration of 1D nanomaterials into microfluidic chips can enable the development of diverse functional microdevices. 1D nanomaterials have been used in applications such as catalysts, electronic instrumentation and sensors for physical parameters or chemical compounds and biomolecules and hence, can be considered as building blocks. Here, we outline and critically discuss promising strategies for microfluidic-assisted synthesis, alignment and various chemical and biochemical applications of 1D nanostructures. In particular, the use of 1D nanostructures for sensing chemical/biological compounds are reviewed.

## 1. Introduction

In the past several years, 1D nanoscale structures including nanowires (NWs), nanotubes (NTs) and nanofibers have drawn great attention due to their superior properties of high surface-to-volume ratio, ultrasmall scale and important applications in nanoscale device generation [1,2,3,4]. Thus, much effort has been devoted to the formation of 1D nanostructures with uniform morphology, using different kinds of precursors, such as inorganic [5], organic [6] or hybrid materials [7]. In general, 1D nanostructures can be formed by top-down and bottom-up approaches. The widely used top-down fabrication methods based on nanolithographic techniques to obtain inorganic nanostructures have the drawbacks of limited choices of materials, expensive instrumentation and time-consuming process [8]. Further development in vapor-phase reactions including chemical vapor deposition or vapor-liquid-solid processes requires high temperature (>500 °C) in the synthesis [9,10]. However, for bottom-up processes, solution-phase reactions are mostly applied, and thus, reactions can be performed under less harsh conditions (e.g., room temperature) for the synthesis of various structures with different kinds of materials. However, the control of reaction and synthesis of uniform 1D nanostructures still remains challenging for bulk bottom-up reactions [2]. Although batch reactors achieved great progress in terms of yield and purity, they all suffer from the poor control over liquid reagents and the positioning of formed nanostructures for further practical applications.

Recent advances have proven that microfluidic systems could provide unique advantages in the bottom-up synthesis of nano- and micrometer scaled structures [11,12]. A microfluidic device is defined as a system that processes or manipulates small volume (10^−9^ to 10^−18^ liters) of fluids, using channels with dimensions of tens to hundreds of micrometers [13]. Compared to bulk methods, microfluidic techniques offer the possibility to highly control ultrasmall amounts of liquid reagents in microchannels. In particular, the reproducible reaction conditions determined by the laminar flow regime make microfluidic advantageous in synthesizing various 1D nanostructures than the conventional large-scale processes [14]. Since 1D nanostructures have shown great potential in building diverse nanodevices, e.g., nanoelectronics, nanooptics, nanosensors, the integration of these ultrasmall structures into microfluidic channels can reveal their full potential for diverse applications [15,16]. In this regard, a microfluidic device can not only enable the controllable alignment of 1D nanostructures by fluid-guided forces, but also be used as a delivery system to supply analytes for sensing applications.

The aim of this review therefore is to deliver a comprehensive overview of the relevant advances in the field of microfluidics for formation and use of 1D nanostructures. The content in this review is organized as follows: in Section 2, recent achievements of the synthesis of 1D nanostructures in microfluidic environments will be summarized. Examples of different synthetic approaches for various classes of materials were presented to introduce the general principles and emphasize the versatility of microfluidics technique; in Section 3, different methods for aligning 1D nanostructures into microfluidic systems are discussed, mainly alignment of pre-grown materials and alignment during the synthesis; the important applications in different fields using various 1D nanomaterials on microfluidic chips will be discussed in Section 4, followed by the conclusions in the Section 5.

## 2. Microfluidic-Based Synthesis of 1D Nanostructures 

The synthesis of 1D nanostructures in microdevices has been studied for more than two decades. In contrast to batch methods, microfluidic techniques can enable the reproducible and well-controlled supply of reagents due to the most important feature of laminar flow conditions in microchannels. To characterize a liquid flow, a Reynolds number (*Re*) is used to describe the ratio of inertial to viscous forces [17]. Microchannels can result in a flow with a low *Re* (less than 2000) which flows laminarly, thereby providing well-defined flow conditions and residence times of compounds. Moreover, interfaces between co-flowing streams can be established and exploited [13]. In addition, microfluidic techniques offer various beneficial possibilities for fluid handling. Functional designs such as mixers, pumps and valves for the handling of small amounts of liquids in the volume ranges down to nanoliter or even picoliter have been integrated in microreactors [18]. In particular, pressure actuated valves can control a reaction by isolating reaction vessels [19,20,21], which is very useful for the localized synthesis of nano- and micro-materials [22]. Besides, temperature in the microchannels can be controlled and stabilized if required since fast heat transfer across the channels walls is feasible to the large surface-to-volume ratio in microchannels, without temperature gradients across the channels’ cross section. The following section will highlight important synthetic approaches for 1D nanostructures in microfluidic systems. An overview of diverse synthesis methods and materials are summarized in Table 1 and will be discussed below in more details. 

### 2.1. Synthesis under Continuous Flow

**Figure 1:**
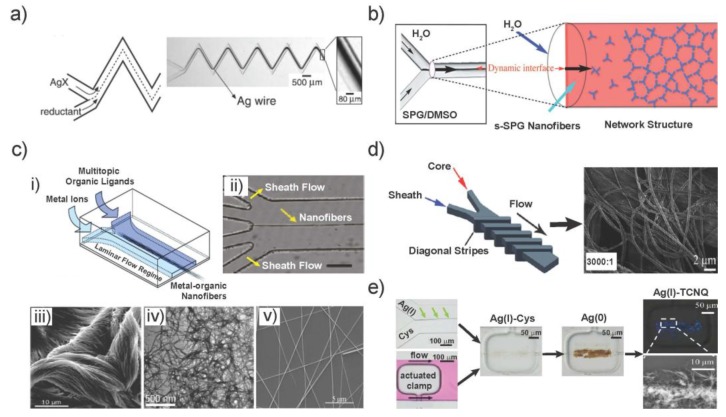
Synthesis of different 1D anisotropic materials using continuous flow method on various microfluidic devices. (**a**) Formation of a silver wire at a fluid-fluid-interface of two co-flowing reagent solutions, a dissolved silver salt and a reductant. (**b**) Mechanism of the formation of the network structure at the dynamic interface; s-SPG nanofibers are continuously supplied to the end of the network structure at the Y-junction point. Thus, the network structure extends along the flow. (**c**) (**i**) Scheme for formation of a metal-organic structure by co-laminar flow of two solutions with either metal or organic precursors. A reaction will take place at the fluid-fluid-interface and result in the formation of a nanoscale structure. (**ii**) Optical microscope images demonstrating the centered assembly along the main channel. (**iii**) SEM image of Cu-Asp nanofiber bundles; (**iv**) TEM image of Ag-Cys nanofibers and (**v**) SEM image of Au-TTF NWs fabricated by the four-inlet microchip. (**d**) Schematics of fluids inside 5-diagonal groove sheath-flow devices (left) and the SEM image of circular PMMA fibers fabricated using this device at sheath-to-core flow-rate ratios of 3000:1; (**e**) Optical microscope images showing the assembly of Ag(I)-Cys at the interface of AgNO_3_ and Cystein aqueous reagent streams (left top); the actuation of a pneumatic clamp in the channel with pink dye flow (left bottom); the formation of Ag(I)-Cys and Ag(0) and Ag(I)-TCNQ NWs (polarized image on right top and SEM image on right bottom). Images in Figure 1 are reproduced with permission (**a**) from [11], (**b**) from [34], (**c**) from [7,14], (**d**) from [12] (The Royal Society of Chemistry) and (**e**) from [38].

Continuous flow reactors provide a constant supply of reagents to form the product along the reactor. A basic microfluidic design which enables continuous coaxial flow reactions has at least two inlets for solutions that meet to build “T- or Y-shape” micromixers. Due to the laminar flow, the reaction occurs at the liquid-liquid interface between the co-flowing precursor solution, which, however, broadens due to diffusion when the fluids and compounds are miscible. In contrast, interfaces of co-flow of immiscible fluids maintain stable. The seminal work of Whitesides and co-workers demonstrated how these interfaces could be exploited for the formation of silver (Ag) microwires, regardless of whether the flow followed a straight or a zigzag-formed microchannel design (Figure 1a) [11].

Later, these simple designs with two or three supply channels were employed to form supramolecular structures by enabling the spontaneous self-assembly of monomers, e.g., hierarchical self-assembled β-1,3-glucan (SPG) networks (Figure 1b) [34] and supramolecular bioorganic (guanosine 5′-monophosphate) nanofiber structures [35].

Continuous flow reactions can also be carried out using additional sheath flows to form hydrodynamic flow-focusing regime which further confines the reaction zone and avoids the direct contact of solutions with the microchannel wall. Our group introduced the microfluidic synthesis of metal-organic 1D nanomaterial on four-inlet microfluidic chips [7,14]. Various metal ions and organic ligands were used to form different metal-organic nanofibers by this method (Figure 1c). The microfluidic synthesis of copper-aspartate (Cu-Asp) could be massively accelerated in the microdevice to occur within microseconds than the bulk reaction. Moreover, silver-cysteine (Ag-Cys) and zinic-4,4′-bipyridine (Zn-4,4′-bipy) were novel types of 1D metal-containing nanofibers that could not be formed in batch synthetic approaches. In addition, the morphology of Au-TTF and other TTF-based metal-organic wires could be tuned by changing the flow rate ratio on the four-inlet microchip, other than the uniform wires formed in bulk [41]. Thus, microfluidic-based synthesis can yield nanoscale metal-organic structures with morphologies and properties that are unequivocally different from those obtained from standard bulk synthesis. 

In an interesting modification, fibers with controlled sizes and shapes were fabricated using hydrodynamic focusing in a microfluidic sheathing device. Straight diagonal or chevron-shaped grooves were integrated in the top and bottom walls of the flow channel to move sheath fluid completely around the core fluid, here to form poly(methylmethacrylate) (PMMA) fibers (Figure 1d) [12]. Portions of the sheath streams were deflected to define the cross-sectional shape of the polymer core. The fiber diameter could be controlled by changing the flow-rate ratio between the sheath and core solutions. In another case, with multi-clamp features integrated into a two-inlet microchip, the localized, stepwise template growth of functional NWs from an amino acid-supported framework was achieved (Figure 1e) [38]. 

### 2.2. Valve-Based Synthesis

**Figure 2:**
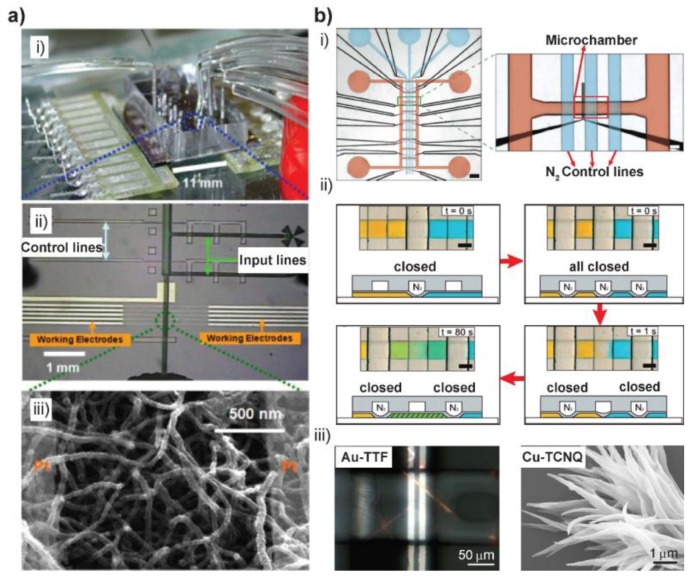
(**a**) Microfluidic device for the synthesis of conducting polymer NWs (CPNWs). (**i**) Actual view of the microfabricted and assembled microreactor. (**ii**) Micrograph of chip design, with microfluidic channel of 16 μm high and 100 μm wide and each of the five pairs of electrode junctions is separated by a 2-μm-wide gap. (**iii**) SEM image of well-defined polyaniline NWs grown in the microfluidic channels. (**b**) Valve-based microchamber arrays for synthesizing metal-organic 1D nanostructures. (**i**) Micrograph of the assembled chip. Black: Pt electrodes, red: fluid layer, blue: control layer. Scale bars: 750 μm (left) and 150 μm (right). (**ii**) The operation of the valves and the diffusive mixing of two food dyes (yellow and blue) in a typical reaction procedure (top and side views). Following the red arrow: supplying of two reagents when the middle valve is closed (t = 0 s); Compartmentalization of two reagent volumes on either side when all three valves closed (t = 0 s); Opening of the central valve to enable the mixing of two solutions (t = 1 s), and finally diffusive mixing (t = 80 s). Scale bars: 100 μm. (**iii**) Polarized micrograph of AuTTF wires formed inside a microchamber (left) and SEM image of Cu-TCNQ obtained from a similar valve-based microchamber device (right). Images in (**a**) are reproduced with permission from [16], (**b**) from [22].

Apart from the continuous flow synthesis, valve-based microfluidic systems which enable reactions in small reactors with subnanoliter volumes are also advantageous in synthesizing 1D nanostrucures. A valve-based system isolates solutions from their supplying continuous flow and provides micro-confinements where reagents can react by diffusion. For example, Tseng´s group developed such a microdevice with controlling lines and a three-electrode system (Figure 2a(i,ii)). By enclosing aqueous solutions of precursors in such confinements, which are equipped with spatially separated platinum electrodes, polyaniline and polypyrrole NWs were form by electrodeposition. The wires bridged the gap between the electrodes and formed an electrical circuit which were used for in situ sensing applications (Figure 2a(iii)) [16,39]. The authors concluded that the flow-free condition and electric field present in the microfluidic confinements appear to be more effective to cause site-specific deposition. Furthermore, the electrodes define the alignment of the structures and no post-formation modification is needed for further application of those in situ formed structures. 

A valve-based two-layer polydimethylsiloxane (PDMS) microfluidic chip was also reported by our group [22] (Figure 2b(i)). By gas pressure on the top control layer, the membrane between the two layers were pressed down to seal channels in the bottom fluid layer. Thus, the control layer was used as valves to control the supplying and reaction of two reagents inside the microchannels (Figure 2b(ii)). The formation of Au-TTF microwires was performed by diffusion-controlled mixing of Au(III) ions and TTF solutions inside the microreactor which was integrated with microelectrodes for further characterization (Figure 2b(iii) left). By firstly depositing an amorphous silver film inside the 300 pL microreactor, Ag-TCNQ nanowires were obtained, following the slow diffusion of TCNQ solution. In a slightly modified design, our group also demonstrated the microfluidic-assisted synthesis of copper-tetracyanoquinodimethane (Cu-TCNQ) nanostructures in ambient environment for the first time (Figure 2b(iii) right) [42].

### 2.3. Other Microfluidic-Assisted Synthesis

**Figure 3:**
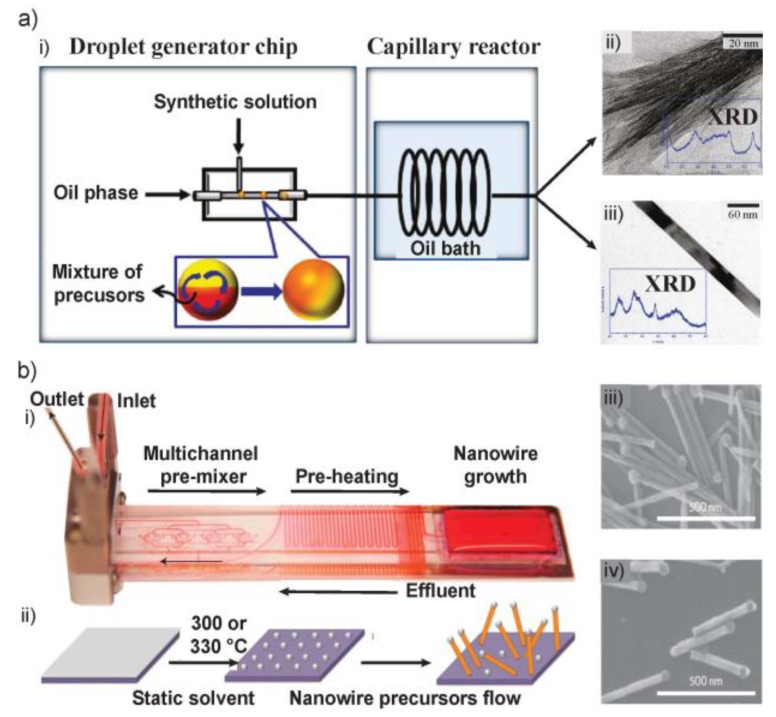
(**a**) Continuous synthesis of inorganic nanomaterials in the DIM system. (**i**) Illustration of the DIM system. TEM images of (**ii**) γ-AlOOH nanofibers and (**iii**) β-FeOOH nanorods. Figure insets in (**ii**,**iii**) showed the XRD patterns of the structures. (**b**) Flow-based high temperature solution–liquid–solid nanowire synthesis. (**i**) Microfluidic device with Rhodamine 6G dye to show different zones. (**ii**) Schematic of synthesis of semiconductor NWs grown from substrates held in flow. (**iii**) SEM images of (**iii**) CdSe and (**iv**) ZnSe NWs grown in flow at 330 °C from 10 nm and 2 nm thick Bi films, respectively. Images in (**a**) are reproduced with permission from [40], and (**b**) from [26].

In contract to the creation of small reactors by means of valves, reaction compartments can be created by use of segmented flow, also referred to as droplet microfluidics. This emerging technology allows the continuous production of various types of nano- and microparticles. For the production of anisotropic structures, it is usually not employed as the inner flow dynamics with two hemispheric flow patterns provides unfavorable conditions for fiber formation. An example of a fast droplet-reaction to form γ-AlOOH nanofibers and β-FeOOH nanorods was presented by Hoang et al., which required only minutes instead of days (Figure 3a) [40]. The proposed droplet- and ionic liquid-assisted microfluidic (DIM) system delineated offer a new synthetic approach for functional but unaccommodating inorganic nanomaterials in a continuous and mild manner. The various dripping and jetting regimes in microfluidic multiphase flows for the synthesis of microfibers have been well compared and reviewed by Nunes et al. recently [43].

Above-mentioned approaches for synthesizing 1D nanomaterials are all performed at low temperature on PDMS microchips. However, the synthesis of semiconductor NWs require high temperature, making it difficult for reaction on normal polymer microfluidic devices. A breakthrough study was the development of a flow-based solution-liquid-solid (SLS) technique which enabled the microfluidic synthesis of cadmium selenide (CdSe) and zinc selenide (ZnSe) structures from a solid substrate (Figure 3c) [26]. 

## 3. Controllable Alignment and Patterning of 1D Nanostructures

Since 1D nanostructures offer great potential as important building blocks for applications in various areas of nanotechnology, the controlled and predictable assembly of nanostructures to form well-ordered structures are highly desirable. Various methods relying only on external forces to align nanostructures have been reported, including electric- or magnetic field induced alignment, and dielectrophoresis [44]. However, these techniques have limitations with respect to the specific materials that can be aligned (charged or ferromagnetic) or the complicated designs of devices (arrays of electrodes). In contrast, microfluidic-guided alignment and patterning approach only uses flow to align 1D nanostructures. Thus, there is no restriction in materials for the alignment and direction by the force generated from the controlled flow. Moreover, the sizes of nanostructures in a simple fluid channel (without pneumatic traps) is not limited since the fluid flow can work on the structures existing in the microchannel, by changing different flow rates. 

Up to now, microfluidic methods applied to assembling 1D nanostructures can be categorized as two different approaches: alignment-after-synthesis and alignment-during-synthesis techniques. The former technique involves the assembly of pre-grown nanostructures, while the latter aligning structures during the synthesis procedure.

### 3.1. Alignment-after-Synthesis

**Figure 4:**
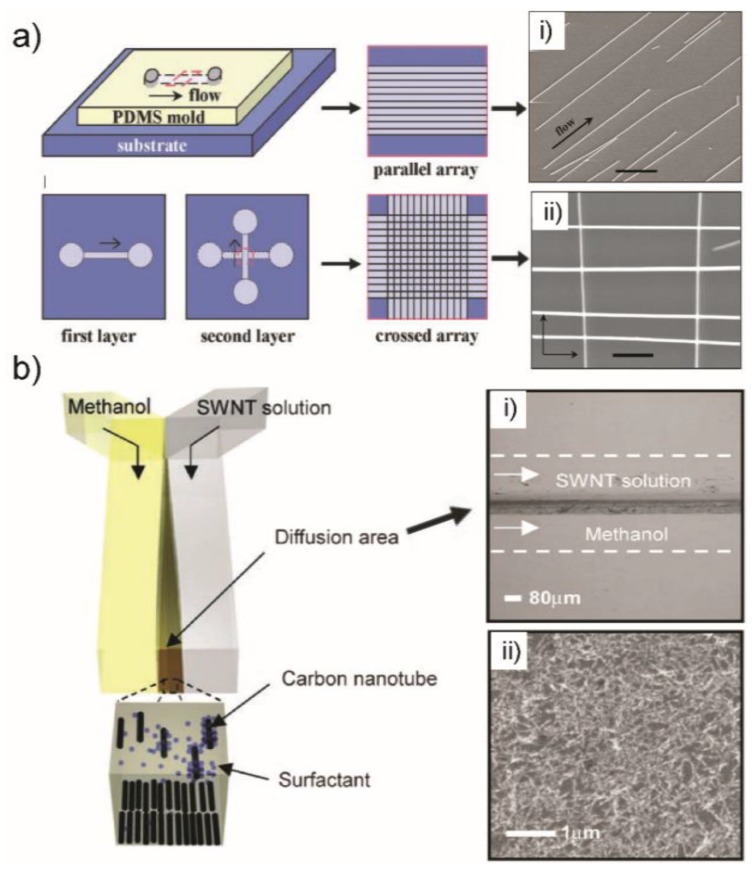
(**a**) Schematic of fluidic channel structures for flow assembly of GaP, InP and SiNW arrays. A channel formed when the PDMS mold was brought in contact with a flat substrate. NW assembly was achieved by flowing the NW suspensions inside the channel. Parallel arrays of NWs are observed in the flow direction on the substrate when the PDMS mold is removed. Crossed NW arrays can be obtained by changing the flow direction sequentially in a second layer assembly process. SEM images of patterned InP NWs in parallel (**i**) and crossed (**ii**) array. (**b**) The microfluidic device with Y-junction for patterning SWNTs by multiphase laminar flow and controlled flocculation. The stripe of SWNTs deposited in the channel (**i**) and the SEM image of this deposition showing the coverage of patterned SWNTs (**ii**). Images in (**a**,**b**) are reproduced with permission from [45,47], respectively.

With the support from the fluidic flow and herewith, the shear forces as the guiding force, NWs and NTs can be aligned and patterned on microchips using respective pre-grown 1D nanostructure solutions. In the hierarchical assembly of NWs (gallium phosphide, GaP; indium phosphide, InP and silicon, Si), the materials were synthesized first and subsequently suspended in ethanol solution [45]. Assembly of NWs was achieved by flowing a NW suspension through microchannels (Figure 4a). Parallel arrays of NWs can be obtained under single flows, with aligned NW arrays in the flow direction (Figure 4a(i)). The flow rates can influence the NW alignment, with faster flow inducing larger shear forces and hence leading to better alignment. In addition, multiple crossed NW arrays were also readily achieved by changing the flow direction sequentially in a layer-by-layer assembly process (Figure 4a(ii)). This approach can be potentially used for organizing other 1D nanostructures into highly integrated device arrays. For example, based on the same technique, high-performance thin-film transistors using oriented SiNW thin films or CdS nanoribbons as semiconducting channels were fabricated [46]. Thus, the shear force alignment offers a simple and general pathway for bottom-up assembly of new electronic and photonic nanosystems.

1D nanostructure can also be patterned with high coverage in confined area of microchannel by controllable reactions and flow conditions. The patterning and depositing SWNTs through the use of controlled flocculation in laminar microfluidic networks was reported [47]. An aqueous suspension of surfactant-stabilized single-walled carbon nanotubes (SWNTs) and methanol that is miscible with water and exhibits a strong affinity for the surfactant were introduced into a microfluidic channel in which fluid flow is laminar (Figure 4b).

The interaction between the two solutions at the liquid-liquid interface leads to the deposition of SWNTs on the solid surfaces (Figure 4b(i)). The orientation of the NTs was determined by the velocity profiles of the local flow fields. The duration of the flows and their velocities as well as the concentration of the SWNT suspension can influence the coverage of pattered structures. This method generates well-defined patterns of aligned SWNT films with controlled density and alignment on a variety of flat and curved surfaces (Figure 4b(ii)) which showed important capabilities in the fabrication of organic electronic devices.

In addition to the pump-driven fluidic flow, the use of capillary forces to align 1D nanostructures was also reported for different kinds of materials. For example, regularly aligned molybdenum selenide infinite NWs were obtained using PDMS molds with micrometer-sized channel network [48]. A droplet (0.1–10 μL) of the molecular wire solution was placed at the open end of the microchannels, and the channels were filled within minutes via capillary action. Owing to the uniaxial flow of the NW suspension restricted by microstructures during solvent evaporation, NWs were thus oriented strictly. Similar approaches have been reported for the alignment of lipid nanotubules [49] and SWCNT [50]. 

The combination of fluidic flows and other external forces have also been reported to assemble specific pre-grown 1D nanostructures. For example, an external magnetic field can induce the trapping of multi-walled carbon nanotubes (MWCNT) while aligning by flow direction [51]. This technique is based on the use of CNTs with a ferromagnetic metal catalyst at one end, which enables the position of the CNTs in microfluidic channel by magnetic field. Then, the shear force from the liquid flow aligns the CNTs parallel to the flow direction in the microchannel [51]. Another example is the simultaneous positioning and orientation of single NWs in a 2D plane in microchannel achieved by using electro-osmotic flow control [52]. An electric field applied across a microfluidic channel creates a flow with velocity directly proportional to the field strength. This flow can move and rotate the immersed single NW due to the viscous stress which depends on the NW´s dimensions and orientations [52]. 

### 3.2. Alignment-during-Synthesis

This technique involves the simultaneous alignment of 1D nanostructures during their formation in microfluidic chips. As shown in Section 2.1, the laminar flow regime which is ubiquitous in continuous flow microfluidics avoids mixing of two adjacent solutions with reagents. The reaction can only occur at the interface between both reagents by diffusion [53]. Consequently, the formation of a structure is flow-directed and aligned. 

**Figure 5:**
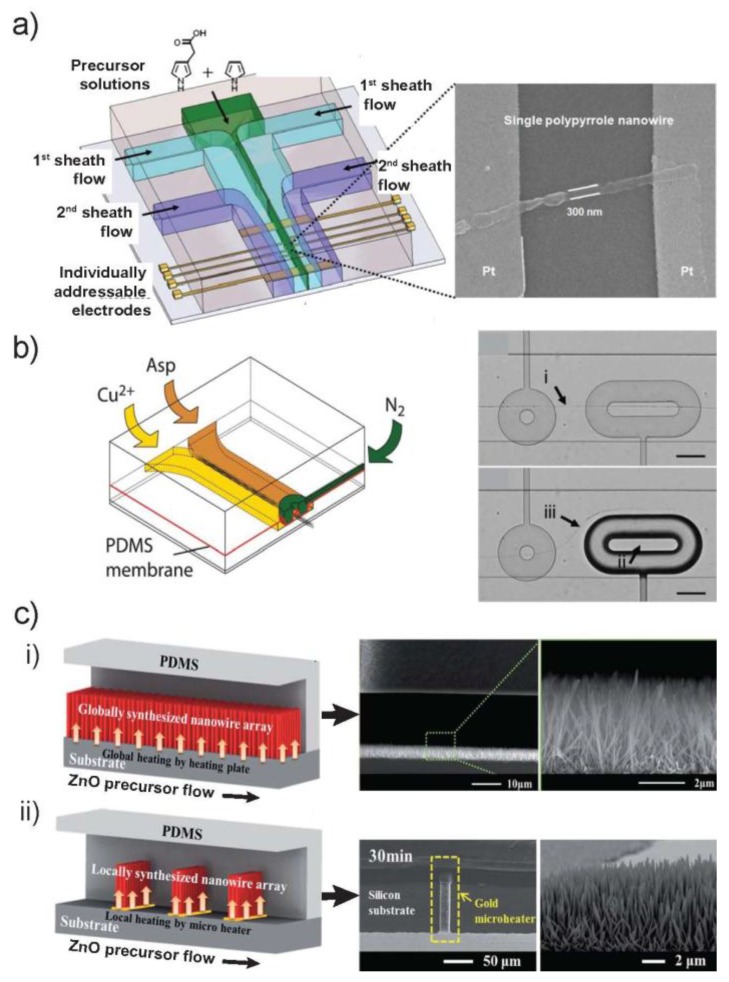
Alignment during synthesis. (**a**) Left: A microfluidic setup with a hydrodynamically focused laminar stream of two precursor solutions is used as a dynamic template for site-specific electrochemical deposition of size controllable conducting polymer micropatterns across individually addressable electrode junction pairs. Right: SEM image of 300-nm-wide Ppy nanopattern across a Pt electrode pair. (**b**) Left: Schematic illustration depicting the principle of formation and trapping of Cu(II)–Asp NW bundles. Right: The NWs are formed at the interface between the two reagent streams (indicated by (**i**)). The position of the interface is determined by the flow rates of the two reagent streams. After actuation of the donut trap, the NWs are embedded inside the donut (**ii**), and a new assembly pathway is established (**iii**). (**c**) Schematics of in situ synthesis and integration of ZnO NWs in microfluidic chip: global synthesis in the entire fluidic channel (left up) and local synthesis by microheaters in the fluidic channel (left bottom). SEM images of ZnO NWs in microchannel: (**i**) globally synthesized (top and cross-sectional view) and (**ii**) locally synthesized by microheater. Images in (**a**) are reproduced with permission from [16], (**b**) from [54] (The Royal Society of Chemistry) and (**c**) from [30] (The Royal Society of Chemistry).

A microfluidic device with hydrodynamically focused laminar stream was reported by Lin et al. (Figure 5a) [16]. This device was employed to obtain width and position controllable solution streams of the monomer precursors by altering the flow rates of sheath streams and the flow rate ratio. Using this microfluidic setting, size-controllable, site-specific electrochemical deposition of conductive single CPNWs (with uniform diameters of 300 nm) across individually addressable electrode junction pairs was achieved (Figure 5a). 

Our group reported the microchip to trap in situ formed bundles of Cu-Asp NWs in microsized clamps, thereby enabling immobilization, positioning and cutting-out of desired lengths [54]. As shown in Figure 5b, the microchip consists of two layers, one of which enables the formation of NWs at the interface of two co-flowing laminar streams (Figure 5b(i)). The other layer, separated by a thin and deflectable PDMS membrane, serves as the pneumatic control layer to impress microsized features (“donuts”) onto the NWs. In this way, a piece of the NW bundle with a prescribed length is immobilized inside the donut (Figure 5b(ii)). The actuation of the donut also enables the emergence of new assembly pathways, providing a tool for dynamically directing assembling processes inside a microchannel (Figure 5b(iii)).

A new and efficient method for the in situ synthesis and integration of NWs within the microfluidic device is hydrothermal reaction. As a low temperature (<100 °C) aqueous process, hydrothermal synthesis is suitable for the liquid phase reaction within the microfluidic device and holds great promise for nanostructure synthesis [55,56]. The direct synthesis and positioning of ZnO NWs within the microfluidic channel, either on the entire substrate (global synthesis) or at selected locations (local synthesis) were demonstrated (Figure 5c) [30]. In this work, ex situ formed ZnO seeds was used to coat a substrate, then PDMS block was bonded to the seeded substrate to produce ZnO nucleation sites within the microfluidic channels. Following, ZnO precursor solution was continuously added, and then the microfluidic system was heated either globally over the whole channel or locally by integrated microheaters, leading to the growth of either global (Figure 5c(i)) or local (Figure 5c(ii)) ZnO NW arrays, respectively. In a methodically comparable synthesis, it was proved that the synthesis rates were five times higher in the microfluidic device than in bulk solution because of the constant replenishing of reagents and clearance of homogenous nucleants that consume reactants in solution [29]. 

In this section, it is well elucidated that microfluidic-based alignment technique is easy-to-handle and cost-effective. Thus, this approach could have great potential in using a wide range of 1D nanomaterials for the fabrication of nanostructure arrays in the future, which is useful for practical applications, e.g., single NW field effect transistor (FET) [57].

## 4. Microfluidic-Assisted Analytical Application of 1D Nanostructures

The integration of 1D nanomaterials with microfluidic devices have enabled the development of various advanced micro devices for different analytical applications. For example, biomolecule enrichment: a capillary microchannel containing TiO_2_-coated ZnO nanorod arrays showed great selectivity, sensitivity and durability for the enrichment of phosphopeptides from tryptic protein digests [58]. A three-dimensional (3D) SnO_2_ nanowire structures embedded in microchannels were ultrafast separation of small biomolecules (such as DNA, protein, and RNA molecules) [59]. Al_2_O_3_ coated 3D SWCNTs networks exhibited nanoparticles filtration and streptavidin capturing capabilities [60]. Another application is cell trapping which uses mechanical traps based on NW pyramidal structures to trap and culture single cells [61]. To enable cell analysis, electrical or mechanical cell lysis by semiconductive CNTs or ZnO NWs arrays [62] embedded in microchannels show their advantages in avoiding cell pollution [63], high-throughput sample preparation [64] and minimized protein damage, compared to conventional techniques [63]. Also, well-aligned diphenylalanine peptide/Pd hybrid NWs in microfluidic channels showed good heterogeneous catalytic performance in hydrogenation and Suzuki coupling reactions [65]. 

Apart from the above-mentioned analytical applications, in the following, we will mainly focus on and review in detail the microfluidic-assisted functional nanosensors. The comparable size of NWs and NTs with biomolecules makes them potentially sense the individual target binding on the surfaces and provides ultrahigh sensitivity, even down to the single molecule level. In addition, microfluidic integration is the critical technique which can forward the nanosensor technology from basic research to real applications. Most of the nanosensors integrated pre-assembled NWs or NTs into a PDMS microfluidic channel first. Afterwards, target chemicals and biomolecules were supplied into the device. Detection of the target analytes can be achieved by changes in signals according to the different readout methods, e.g., electrical and optical techniques. 

### 4.1. Nanowire Sensors

The most widely applied nanowire substrate for sensor is SiNWs. The preference of SiNWs as a popular sensor substrate can be attributed to their ultrasmall sizes and the controllable dopant type [66]. The label-free sensing possibility by electrical readout. This kind of sensor based on semiconductive 1D nanostructures is also called FET sensor, which functions by transduction of analyte binding on FET surface into changes in current-voltage signals between the source and drain electrodes [67]. Thus, the binding of analytes can be monitored by a direct change in conductance or related electrical property, allowing the label-free detection of target molecules [68]. 

The pioneering work used a boron-doped Si NW for real time pH monitoring, small biospecies sensing, as well as immunoassay [72]. Since then, many research groups have employed SiNW FET to build biosensors and successful solution-phase sensing has been demonstrated for small molecules [73], antibodies [74], viruses [75], cardiac biomarkers [76], DNA [77,78,79] and proteins [15]. These high sensitive, real-time and label-free detection biomolecules in real samples like human serum using SiNW-based immunosensor [80] can provide useful information for developing diagnostic techniques. Apart from electrical sensors, NWs were also proven to act as optical sensors, which was an attractive sensing technique, especially for multiplexed analysis on a single microfluidic platform [81]. In a microsystem that integrating protein micropatterns on vertically aligned SiNWs into microfluidic devices for immunoassays [82], high fluorescent signals were observed after the specific antibody-antigen binding. 

**Figure 6:**
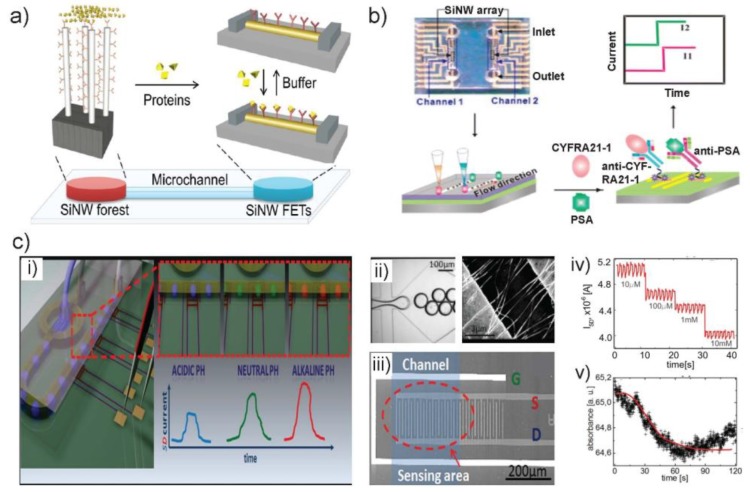
(**a**) Scheme of the operation of a whole-SiNW selective filtering and sensing device on a single chip platform. (**b**) Image of PDMS microfluidic system superimposed on SiNW arrays for fluid exchange (left top). Scheme of flowing antigens through the PDMS microchannel (left bottom); rapid recognition of cytokeratin 19 fragment (CYFRA21-1) and prostate specific antigen (PSA) using microfluidic integrated SiNW biosensor (right bottom) and real-time current readout (right top). (**c**) Compact NW sensors probe microdroplets. (**i**) Concept illustration of the integration of a SiNW FET into a droplet microfluidic system. Droplets with different pH will give rise to a different conductance change. (**ii**) Multiphase microfluidics, in which two immiscible liquids are combined to create identical isolated droplets (left) and SEM picture of the SiNW connecting source and drain electrodes. (**iii**) Top view of a single ion-sensitive field effect transistors (ISFET), showing source (S), drain (D), and the Ag/AgCl reference electrode (G) on silicon wafer. (**iv**) Raw current signal showing the decrease with increasing concentrations of PBS. (**v**) The changes in absorbance signal during GOx enzymatic oxidation of glucose confirmed the parallel readout of the reaction droplets using integrated optical detection setup. Images in (**a**) are reproduced with permission from [69], (**b**) from [70] and (**c**) from [71].

Although the above-mentioned nano-biosensors based on SiNW or NW arrays have proved the efficiency, selectivity and sensitivity in detecting various bioanalytes, advanced microdevices are still highly demanded to achieve the high throughput analysis, low limit of detection and diagnostic applications. Thus, the development of multifunctional devices based on SiNWs has become a new research focus in recent years. Research efforts lie mainly in three aspects: Firstly, to develop total integrated microsystem with multi-applications on the chip platform. The first demonstration is an all-NWs device for the whole direct analysis of blood samples on a single chip (Figure 6a) [69]. This modified NW-based device was able to selectively collect and separate specific low abundant proteins, while easily removing unwanted blood components (proteins, cells) and achieving desalting effects, without the requirement of time-consuming centrifugation steps and the use of desalting or affinity columns. Furthermore, the SiNW forest-based sensors arrays was used for the real-time, label-free and ultrasensitive detection of protein biomarkers directly from blood samples in less than 10 min [69].

The second research effort is to develop a method for the simultaneous detection of multi-analytes. One of the first immunosensors reported was based on SiNWs and used for the detection of different antigens by SiNW-based FETs arrays [83]. A two-channel PDMS microfluidic integrated CMOS-compatible SiNW FET arrays for label-free and ultrasensitive electrical detection of cancer biomarkers was reported (Figure 6b) [70]. The NW arrays showed ultrahigh sensitivity of different antigens with detection to 1 fg/mL in buffer solution and high selectivity towards other similar cancer biomarkers. In addition, a similar device with dual CMOS polysilicon NW sensors for simultaneous detection of multiple analytes and on-chip whole blood processing was developed [84].

A third research focus is the multi-readout technique e.g., electrical and optical detection on the same sensing device, which is used for verifying the sensing result and eliminating ineffective signal. A droplet microfluidic system with the compact SiNW FET which was used for in-flow electrical detection of each individual droplets was reported (Figure 6c) [71]. By monitoring the changes of the source-drain current (I_SD_), the authors succeeded in chemically probing the content of numerous (~10^4^) droplets independently and resolving the pH values and ionic strengths of the encapsulated solution. They also demonstrated the glucose oxidase (GOx) assay in droplets, measured by SiNW FET sensor and integrated optical spectrometer in parallel. Such realization of dual detection (SiNW sensor and optical luminescence) is reported for the first time.

Conductive SiNWs can also be used to probe flow velocity in microchannel. A first example was demonstrated by using SiNWs as sensitive probes for streaming potential, flow velocity and ionic strength, which also opened up the new applications of SiNWs in microfluidics [85]. Additionally, nanowires based on other metal, organic or inorganic materials also showed their benefits in building functional microdevices. For example, sensors fabricated with indium arsenide (InAs) nanowire [86] or nanocilia [87] showed their abilities for fast responding to fluids inside microchannels. Polypyrrole NW-based FET [88] and vertical germanium NW array [89] were reported for their excellent and tunable sensitivity towards pH variations in microfluidic channels. In situ synthesized Au-TTF metal-organic wires showed the sensing abilities to catecholamines and antigens after different surface functionalization [90]. Inorganic silica NWs were developed into a multifunctional optical sensor that utilized the evanescent field of a subwavelength NW waveguide to perform optical spectroscopy on femtoliter probe volumes [91]. Each sensor is capable of carrying out absorbance, fluorescence, and SERS measurements on the same analyte while operating within a microfluidic flow cell.

### 4.2. Nanotube Sensors

The mostly studied nanotube is CNTs, due to the nanoscale sizes and high electrical conductivity that make them ideal substrates for fabricating electrochemical nanosensors. CNTs, the tubular analogy of graphene, can be divided into SWCNTs and MWCNTs, according to the different number of graphene sheets on the structures. SWCNTs are well known for their semi-conductive or metallic properties, depending on the specific arrangement of carbon atoms, while MWCNTs tend to behave like metals [92,93]. These different electronic properties have induced diverse applications of these two CNTs, with MWCNTs mainly used as microelectrodes in electrochemical sensors [94], while SWCNTs mostly behaved as transistors [95] but also used for microelectrodes in an electrochemical cell [96].

**Figure 7:**
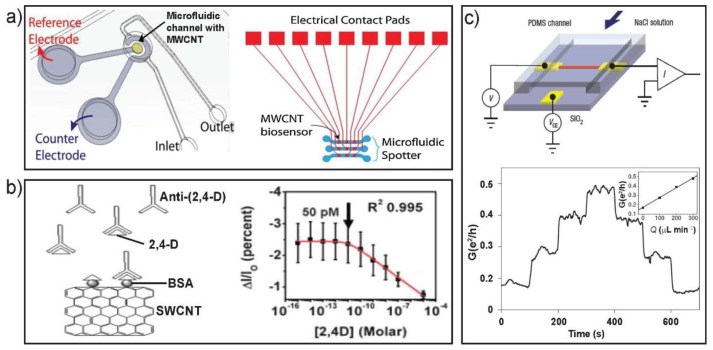
Microfluidic MWCNT-EC (electrochemical) detection system including (**a**) PDMS microfluidic channel with Pt counter and Ag/AgCl reference electrodes (left). The design of microfluidic biomolecule spotter with integrated MWCNT chip (right). (**b**) Schematic of direct assay detection of free 2,4-D (left) and response of the SWCNT-FET showing the response of the sensor toward BSA and anti-(2,4-D). (**c**) Experimental setup for the NT-based flow sensor on a PDMS microfluidic chip (top) and conductance-time signal for the flow sensor with flow rate stepped up sequentially through 0, 100, 200 and 300 μL min^−1^, and then back down sequentially through the same values (bottom). Images in (**a**) are reproduced with permission from [100], (**b**) from [102] and (**c**) from [103].

In an electrochemical sensor, typically, a three-electrode-system is involved: a working electrode based on CNTs, where the reaction takes place; a counter electrode and a reference electrode (Figure 7a). The nanosensors respond to the chemical reaction of oxidizable or reducible substance that causes electron transfer on the electrodes, and show signals proportionally related to the concentration of analytes on the surface of the working electrode [81]. The first capillary electrophoresis microchip using MWCNT-modified working electrode as the electrochemical detector was applied to the detection of several analyte classes [97]. In addition, other studies on the modification of different electrode substrates with MWCNT were used for sensitively detecting various small biomolecules (antioxidants) [98,99,100], as well as antigens (PSA) [101] on microfluidic chips. Notably, the electrochemical immunosensor showed faster and more sensitive quantification of PSA in human serum samples than standard spectrophotometric detection method [101].

As in the case of SiNW FETs, immunosensors based on SWCNT FETs have also been extensively investigated [104]. The difference between SiNWs-based and SWCNT-based immunosensors is that the former need covalent binding of receptors on the NW surface, while the latter can be built by either non-covalent [105] or covalent immobilization [106] on the CNT surface. When using non-covalent approaches, bifunctional linkers that have both hydrophobic and hydrophilic ends would be introduced to bridge CNTs and receptors to keep the sensor stable [107]. As to direct covalent binding of receptors on CNTs, functional groups will be introduced to CNT sidewalls to allow covalent immobilization [108]. Such an immunosensor was produced and used to perform direct, indirect and competitive immunoassays for the real-time and label-free detection of 2,4-dicholorophenoxy acetic acid herbicide at fetomolar level on a microfluidic platform (Figure 7b) [102]. Apart from immunosensors, a variety of biological targets like DNA [77], cholesterol [109] and glucose [110] could be monitored using functionalized SWCNTs as sensors. In the development of various nanoFET sensors, conductive nanostructures including metal oxide and polymers were also demonstrated to show good performance in biosensing [102].

CNTs have also been demonstrated to sense the chemical potential of solutions containing redox-active molecules [96,111]. The leading work in NT flow sensors was based on bulk SWCNTs [112]. Afterwards, many studies showed the flow sensing ability of CNT transistors [95,113]. However, only after the demonstration of individual CNT transistors in microscale channels, that a novel approach for investigating electrokinetic phenomena and fluid dynamics at the nanometer scale was developed (Figure 7c) [103]. Compared to other flow sensor composed of Si NWs [85], CNTs was low-cost and more effective, having potential development of a paper-based microfluidic sensor device. 

NTs made of other materials, e.g., noble metals, show optical sensing possibilities in microfluidic systems based on surface enhanced Raman spectroscopy (SERS) [114]. An optofluidic SERS device using Au/Ag/Au nanotubes as substrates showed the success in on-chip detection of vasopressin [115].

## 5. Conclusions 

This review presents an overview of recent advances in microfluidics-based synthesis, alignment and use as sensor of various 1D nanostructures. The combination of microfluidics and micro- and nanostructures has led to significant advantages in various fields. The unique features of microfluidics, especially the laminar flow, the high surface-to-volume ratio, the enhanced mass transport and heat transfer were used very efficiently to influence reactions and control structure formation. Numerous examples proved the advantages of microfluidic synthesis vs. bulk synthesis for 1D structure formation. Although the integration of 1D nanostructures with microfluidics have achieved great progress, there are still many challenges in the synthesis, alignment and application. For example, it is still difficult to use microfluidic-assisted techniques to form diverse structures, i.e., to precisely control the morphology, dimension (length, diameter) or the nucleation site. 

In addition, the fabrication and operation of the microfluidic devices requires expertise and training. To make these approaches accessible to non-specialists, significant simplifications in the handling are required. This is particularly important since the presented 1D nanosensensors are typically not suitable for multiple uses. Cheap frames to immobilize and connect sensors and interface the nano-sized structures with micro-/millimeter connectors would be helpful in this regard.

For sensing applications, multifunctional devices with high sensitivity and fast response are demanded. With respect to biological analyses or diagnostic applications where the sample is, e.g., blood and other body fluids, the major challenge is the limited specificity of the sensors. Currently, the sample must be processed before detection (e.g., purification, separation), or the nanostructures must be functionalized with specific molecules like antibodies to capture the analytes from a mixture. Due to the high surface to volume ratio of 1D nanostructures, a high number of antibodies or other capture molecules can be theoretically immobilized on the nanostructure, which may increase sensitivity due to the high accumulation of the analyte at the 1D structure. However, such molecules need specific conditions (temperature, pH, buffers) and the final detection of the analyte still relies typically on fluorescence, bioluminescence or electrochemical assays and hence shows no significant improvement over surface-bound capture proteins. In other words, the increased efforts in making 1D structures is still not justified for these more complex applications. Currently, 1D structures show the most promising results when electrical/electrochemical signals are recorded, where charged molecules or polar gases are detected or pH values are measured. 

An exciting prospect is the possibility to transport nanowires into cells and organs where they may sense parameters like temperature or pressure, or ultimately selected molecules. Here, the uptake of the nanowires across the cell membrane as well as toxicity and biocompatibility of the material is of importance. In any case, we believe that many challenges will be solved in future and nanofibers and nanowires will become more important for sensing in ultrasmall volumes.

## Figures and Tables

**Table 1:** Summary of selected microfluidic-synthesized 1D nano-/microstructures.

Microfluidic Method	Class of Material	Composition	Synthetic Approach	Morphology	Ref.
Continuous flow (Coaxial flow or Hydrodynamic flow focussing)	Inorganic	Alginate	Ionic crosslinking	Microfiber	[23,24,25]
		Cadmium selenide; Zinc selenide	Solution-liquid-solid growth	Nanowire	[26]
		Copper-carbon	Hydrothermal growth	Nanowire	[27,28]
		Silver	Self-assembly	Microwire	[11]
		Zinic oxide (ZnO)	Hydrothermal, ZnO seed	Nanowire	[29,30]
	Organic	4-Hydroxybutyl acrylate; Poly(ethylene glycol) diacrylate	Photo-polymerization	Microfiber/microtube	[6,31]
		Polyacrylonitrile; Polysulfone; Polystyrene; Poly(methylmethacrylate)	Solvent-extraction hardening	Nano-/microfiber	[12,32]
		Poly(lactic-*co*-glycolic acid)	Solvent-exchange solidification	Microfiber	[33]
	Bio-organic	B-1,3-glucan; Guanosine 5′-monophosphate	Self-assembly	Nanofiber	[34,35]
		Chitosan	Ionic crosslinking	Microfiber/microtube	[36,37]
	Metal-organic	Copper-aspartate; Silver-cysteine; Zinic-4,4′-bipyridine	Self-assembly	Nanofiber	[14]
		Gold-tetrathiafulvalene (Au-TTF)	Self-assembly	Nano-/microwire	[7]
		Silver-tetracyanoquinodimethane (Ag-TCNQ)	Self-assembly	Nanowire	[38]
Valve-based	Organic	Polyaniline/polypyrrole	Electro-polymerization	Nanowire	[39]
	Metal-organic	Au-TTF/Ag-TCNQ	Self-assembly	Microwire	[22]
		Copper-tetracyanoquinodimethane	Self-assembly	Nano-/microwire	[22]
Microdroplet	Inorganic	γ-AlOOH/β-FeOOH	Ion liquid template	Nanofiber/nanorod	[40]
